# Malaria in England

**Published:** 1902-05-24

**Authors:** 


### MALARIA IN ENGLAND.

The probabilities are that the malaria plasmodium has died out in England and that practitioners in this country need not now expect to meet with cases of indigenous malaria. On the other hand, owing to the increased facilities for travel, we come across far more people than formerly who have been exposed to malaria abroad, so that its potentiality as a cause of fever must always be borne in mind. At the last meeting of the Medical Society, Dr. Patrick Manson said that although there were many clinical signs of malaria, there was only one clinical symptom that justified a positive diagnosis, namely, periodicity—tertian or quartan—a rhythmical recurrence of symptoms every 48 or 72 hours. Quotidian periodicity was, he said, valueless in diagnosis. Multiple infection by two kinds of parasites or by two or three generations of the same kind of parasite might give rise to double tertian or treble quartan infection with diurnal recurrence of symptons, but this was not a true quotidian periodicity. A similar periodicity was usual in almost all febrile complaints, and to make quotidian periodicity a ground for diagnosis was wrong and dangerous and was, perhaps, the commonest cause of erroneous diagnosis. Diagnosis might also be made by the administration of quinine, for, whatever might be the case in tropical countries, quinine, properly given, never failed to arrest a malarial attack in England within 48 or at most 96 hours of its administration. A periodic remitting fever, therefore, which persisted after adequate dosing with quinine—10 grains of the drug three times a day for four days—was not malarial. Diagnosis by the microscope was, however, the most satisfactory method if undertaken by one with experience. But a negative result by an amateur was valueless, while a positive result was not much more to be trusted. In regard to the administration of quinine not only must the dose be adequate, but it must be so given that it shall be absorbed. A catarrhal condition of the mucous membrane was usual in severe malarial attacks, and such a condition was very unfavourable to absorption, so that in these cases the quinine ought to be given hypodermically or by enema. To give it in the tablet or coated pill form was a most dangerous method in severe cases, and most unsatisfactory for purposes of diagnosis. Another interesting statement was made by Dr. Manson in regard to the length of time that an infection with malaria can persist. This he placed at three years, and if we put this side by side with his other statement as to indigenous malaria being a thing of the past in England, we may draw the important conclusion that a case of fever in a patient who has not been in a malarious country for three years or more is not a case of malaria.

